# Effect of mindfulness breathing versus acupoint stimulation on anxiety, fatigue, and sleep quality among children undergoing stem cell transplantation: a three-arm randomized controlled trial

**DOI:** 10.3389/fmed.2026.1952564

**Published:** 2026-10-14

**Authors:** Nagwa Ramadan Esmail Magor, Abdelaziz Hendy, Samar Eldesoky Mohamed Ads, Sahar Wasfy Mahmoud Melika, Sahar Mahmoud Elkhedr Abdelgawad, Rasha Kadri Ibrahim, Randa Alsunitan, Mohamed Ramadan Elshanshory, Noha Fawzy Mohamed Fonon, Seham Eid Hashem Elhalafawy

**Affiliations:** 1 Pediatric Nursing Department, Faculty of Nursing, Tanta University, Tanta, Egypt; 2 Department of Maternal and Child Health Nursing, College of Nursing, Qassim University, Buraydah, Saudi Arabia; 3 Pediatric Nursing Department, Faculty of Nursing, Ain Shams University, Cairo, Egypt; 4 Pediatric Nursing Department, Faculty of Nursing, Kafr El-Sheikh University, Kafr El-Sheikh, Egypt; 5 Nursing Department, Fatima College of Health Sciences, Madinat Zayed, Al Dhafra Region, United Arab Emirates; 6 Pediatric Nursing Department, Faculty of Nursing, Damanhur University, Damanhur, Egypt; 7 Department Pediatric Hematology/Oncology, Faculty of Medicine, Tanta University, Tanta, Egypt; 8 Neuropsychiatry Department, Faculty of Medicine, Tanta University, Tanta, Egypt

**Keywords:** acupoint stimulation, anxiety, fatigue, hematopoietic stem cell transplantation, mindfulness breathing, pediatric oncology, sleep quality

## Abstract

**Background:**

Children undergoing hematopoietic stem cell transplantation (HSCT) commonly experience anxiety, fatigue, and sleep disturbances, which may negatively affect their quality of life and recovery. Non-pharmacological interventions, including mindfulness breathing and acupoint stimulation, have emerged as supportive approaches for symptom management.

**Aim:**

This study evaluated and compared the effects of mindfulness breathing, acupoint stimulation, and routine care on anxiety, fatigue, and sleep quality among children undergoing HSCT.

**Methods:**

A three-arm randomized controlled trial was conducted at the stem cell transplantation unit of Tanta University Hospitals, Egypt. Ninety children aged 7–17 years were randomly assigned to three equal groups: control (routine care), mindfulness breathing, and acupoint stimulation. The assigned interventions were delivered once daily for seven consecutive days before transplantation and continued for one month after transplantation. Anxiety, fatigue, and sleep disturbance were assessed using the State-Trait Anxiety Inventory for Children, Fatigue Severity Scale, and PROMIS Parent Proxy Sleep Disturbance Scale, respectively. Assessments were conducted at baseline, one week, and one month after intervention.

**Results:**

Baseline sociodemographic and clinical characteristics were comparable among the three groups. Significant Group×Time interactions were observed for diastolic blood pressure, respiratory rate, pulse rate, anxiety, fatigue severity, and sleep disturbance scores (*p* < 0.001). Both mindfulness breathing and acupoint stimulation resulted in significant improvements compared with routine care. Mindfulness breathing demonstrated greater improvements across several physiological and psychological outcomes compared with acupoint stimulation and routine care.

**Conclusion:**

Mindfulness breathing and acupoint stimulation are promising complementary interventions for improving physiological and psychological outcomes among children undergoing HSCT. Mindfulness breathing showed greater improvements across several outcomes and may represent a useful supportive nursing intervention during the transplantation period. Further multicenter studies with longer follow-up are recommended to confirm the sustained effects of these interventions.

**Clinical Trial Registration:**

The Pan African Clinical Trial Registry PACTR 202407894821147. Date: 17 July 2024. www.http://pactr.samrc.ac.za

## Introduction

Hematopoietic stem cell transplantation (HSCT) is a therapeutic approach for different childhood cancers, blood disorders, and other conditions. HSCT has some risks and challenges. Because of long and complex treatment, childhood disorders require isolation and put children at risk for transplantation-related complications (1–3). Treatment is further divided into different phases, and each of them has its own challenges; for example, in the phase of transplantation, children feel anxiety. Children go through different emotional burdens because of treatment and its subsequent aftereffects, which affect children's social and psychological well-being. Quality of life is also impacted because of treatment. It is well documented that HSCT has a broad range of psychosocial effects on children (4, 5).

Children undergoing HSCT may experience substantial psychological and physical burdens throughout the transplantation process. Anxiety can occur before and during hospitalization because of uncertainty regarding the procedure, its potential risks, and possible long-term consequences (6, 7). Following transplantation, fatigue is also common and may persist for months or even years, negatively affecting children's cognitive functioning, emotional well-being, concentration, and overall quality of life (8–10). In addition, children may experience other psychological symptoms, including depression, irritability, and discomfort, during and after HSCT (9–11). These findings highlight the need for supportive interventions that address both psychological distress and treatment-related symptoms throughout the transplantation period.

Recently, increasing attention has been directed toward non-pharmacological interventions as supportive approaches for reducing treatment-related symptoms among children undergoing cancer therapies and HSCT. These approaches include relaxation techniques, mindfulness practices, massage therapy, and acupoint stimulation (12). Such interventions may provide safe, accessible, and complementary strategies to enhance symptom management alongside conventional medical care.

Mindfulness practices require the individual to purposefully recognize and accept sensory experiences, feelings, and thoughts that are ongoing in the present (13). These may include sensations of movement and/or breathing. Other practices may incorporate the use of imagery and visualizations (14, 15). Although the practices may vary, the goal is for the individual to strengthen awareness and self-regulation of sensory experiences. Improved self-regulation of emotions may allow the child to better manage and self-direct behaviors during medical interactions (16, 17).

There is accumulating evidence to suggest the use of mindfulness-based interventions to manage anxiety and improve the quality of life of children diagnosed with cancer. Other symptoms of stress and anxiety may be reduced through the use of these practices, as well (18). Because the symptoms of mindfulness practices lend themselves well to management of a number of side effects of treatment for cancers, as well as HSCT, further study to validate these effects is warranted (14, 19).

Acupoint stimulation, particularly acupressure, is another complementary non-pharmacological approach that has demonstrated potential benefits in reducing anxiety, improving sleep quality, and alleviating fatigue-related symptoms. Acupressure is based on the principles of Traditional Chinese Medicine and involves applying pressure to specific acupoints using fingers or specialized devices to promote relaxation and symptom relief (20). Compared with acupuncture, acupressure is non-invasive, does not involve needles, and can be easily implemented in clinical settings or taught for self-management (21).

Several acupoints have been associated with symptom regulation, including Shenmen (HT7), which has been frequently used for sleep-related problems; Yintang (EX-HN3), which has been associated with relaxation and anxiety reduction; and Zusanli (ST36), which has been investigated for fatigue management (22, 23). Previous studies have suggested that acupressure may influence autonomic nervous system regulation and neurotransmitter activity, contributing to reductions in anxiety and improvements in sleep and emotional balance (20, 23).

Pediatric nurses assist children and families during HSCT by evaluating and managing symptoms and providing teaching. Using their expertise in assessment and intervention, pediatric nurses help develop safe, feasible, and innovative nursing interventions during the transplant process to improve children's symptom control and quality of life (24). Based on the available evidence, the use of mindfulness breathing and acupoint stimulation during HSCT has shown positive effects. However, more evidence is needed to draw conclusions on the relative effects of these interventions for this population.

The aim of the present study is to evaluate and compare the effectiveness of mindfulness breathing, acupoint stimulation, and routine care on anxiety, fatigue, and sleep quality among children undergoing stem cell transplantation.

### Research hypotheses

H1: Children undergoing stem cell transplantation who receive mindfulness breathing or acupoint stimulation will demonstrate lower anxiety and fatigue levels and improved sleep quality compared with children receiving routine care.
H2: Mindfulness breathing will produce greater improvements in anxiety, fatigue severity, and sleep quality compared with acupoint stimulation and routine care.

### Subject and methods

#### Research design

This randomized controlled trial was designed, conducted, and reported in accordance with the Consolidated Standards of Reporting Trials (CONSORT) 2025 guidelines for parallel-group randomized trials. The study adhered to the recommended checklist to ensure transparency, reproducibility, and methodological rigor in reporting all aspects of trial design, implementation, analysis, and interpretation.

The present study used a three-arm parallel-group randomized controlled trial design with a 1:1:1 allocation ratio. Participants were randomly assigned to one of three groups: a control group (routine care), a mindfulness breathing group, and an acupoint stimulation group.

Trial Registration: The Pan African Clinical Trial Registry Identifier registered this trial (PACTR 202407894821147). 17 July 2024. https://www.http://pactr.samrc.ac.za

#### Setting

The study was conducted at the stem cell unit of Tanta University Hospitals in El-Gharbia, Egypt, which is affiliated with the Ministry of Higher Education and Scientific Research.

#### Participants

We enrolled 90 children in this randomized controlled trial and randomly assigned them in a 1:1:1 ratio to three equal groups (*n* = 30 each): the control group, which received the routine care provided by the stem cell unit; the mindfulness breathing group, which received mindfulness breathing exercises in addition to routine care; and the acupoint stimulation group, which received acupoint stimulation alongside routine care. Randomization was performed using a computer-generated random number sequence prepared by an independent researcher who was not involved in participant recruitment or intervention procedures. Participants were recruited and enrolled by the research team, and group allocation was determined by opening the next sequentially numbered, opaque, sealed envelope (SNOSE) after participant enrollment. Each envelope contained the assigned group allocation, ensuring allocation concealment and preventing foreknowledge of group assignment. All interventions were delivered by trained researchers according to standardized protocols.

#### Sample size

The sample size was calculated using G*Power software (version 3.1.9.7) (25). Because the primary aim was to compare the effectiveness of three parallel groups (control, mindfulness breathing, and acupoint stimulation), the initial sample size estimation was based on a one-way ANOVA model for detecting between-group differences, assuming a medium effect size (f = 0.25), a significance level of 0.05, and a statistical power of 0.80. The calculated sample size was adjusted to account for potential attrition and incomplete data, resulting in a final sample of 90 children (30 participants per group). The repeated longitudinal measurements were subsequently analyzed using repeated-measures ANOVA/linear mixed models to evaluate within-subject changes over time and the Group×Time interaction effect. We adjusted the estimated required sample size to account for possible attrition, incomplete data, or non-compliance during the intervention period. Accordingly, the final sample consisted of 90 children, with 30 participants allocated to each group (control, mindfulness breathing, and acupoint stimulation).

### Inclusion and exclusion criteria

Children were included in the study if they were aged between 7 and 17 years, diagnosed with hematological or oncological diseases, and admitted for hematopoietic stem cell transplantation during the study period. Eligible children included those undergoing their first or subsequent stem cell transplantation, who were clinically stable and able to participate in the intervention sessions. Participants were required to be able to communicate and understand the instructions related to mindfulness breathing and acupoint stimulation, with informed consent obtained from their parents or legal guardians and assent from the children when applicable.

Children were excluded if they had severe cognitive impairment, developmental delay, or communication difficulties that interfered with participation or completion of assessment tools. Children with unstable medical conditions requiring intensive care or emergency management, severe neurological or psychiatric disorders, or conditions that could affect their ability to participate in the intervention were also excluded. Additionally, we excluded children with skin lesions, local infection, or medical contraindications to acupoint stimulation; those receiving treatments that could significantly affect anxiety, fatigue, sleep, or physiological parameters; and those unable to complete the intervention sessions or follow-up assessments. Withdrawal of parental consent or the child's refusal to participate at any stage was also an exclusion criterion.

### Participant flow (CONSORT flow description)

A total of 90 eligible children were enrolled and randomly allocated equally into three groups (*n* = 30 per group). All participants received their allocated interventions and were followed up at 1 week and 1 month post-intervention. There were no reported losses to follow-up or exclusions after randomization. All randomized participants were included in the final analysis. (A CONSORT flow diagram illustrating participant recruitment, allocation, follow-up, and analysis is presented in Figure 1.).

**Figure 1:**
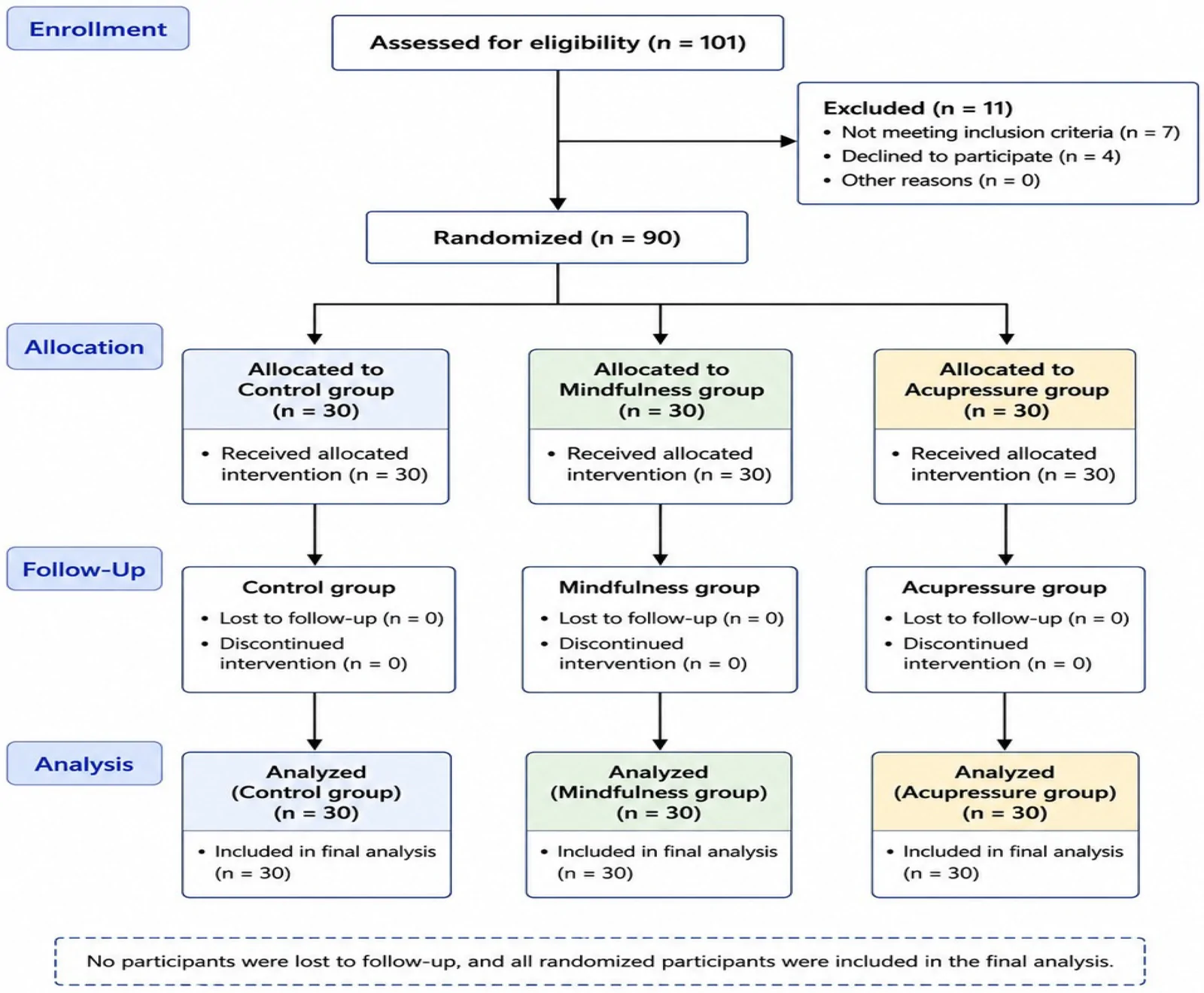
Consort flow diagram of participant recruitment, allocation, follow-up, and analysis.

#### Tools

Tool I: Children's biosocio-demographic data were developed by researchers after reviewing the related recent literature (2, 19). This tool included two parts as follows:

Part A: Sociodemographic data of children such as age, gender, birth order, educational status, and residence.
Part B: Biomedical data, including duration of hospitalization, comorbidities, and medical diagnosis. In addition, transplant data will include transplant type and number, stem cell source, and preparation regimen. Physiological indicators of children: blood pressure, pulse, and breathing rate.

Tool II: State-Trait Anxiety Inventory for Children (STAIC-S): Spielberger (1973) (26) developed it, and the researchers adopted it to assess state anxiety in children. It consists of 20 sentences related to children's feelings at a particular time. There are direct sentences (2, 4, 5, 7, 9, 11, 15, 16, 18, and 19) that indicate negative emotions, and reverse-scored sentences (1, 3, 6, 8, 10, 12, 13, 14, 17, and 20) indicate positive emotions. Children are asked to rate their agreement on a 3-point scale using the stem “I feel…” for 20 sentences, both indicative of the presence of anxiety (e.g., 1 = not upset; 2 = upset; 3 = very upset) and for the reverse-worded items (e.g., 1 = very calm; 2 = calm; 3 = not calm). Total scores are the sum of the item scores, ranging from 20 to 60. For statistical purposes: Low anxiety from 20 to 30, average anxiety from 31 to 40, and above average anxiety from 41 to 50. A score between 51 and 60 indicates a very high level of anxiety.

Tool III: Fatigue Severity Scale (FSS): The FSS questionnaire was developed by Krupp et al. (1989) (27). It was Likert-scaled to assess the severity of fatigue symptoms experienced by the children and how it affected their activities over the past 7 days, and it contained nine statements. Each statement was scored on a scale from 1 to 7. A low value (1) indicated strong disagreement with the statement, while a high value (7) indicated strong agreement. Higher scores indicate more severe fatigue and greater impact on the child's activities. The total score was the sum of the items' scores. Mild level of fatigue from 9 to 36, moderate level of fatigue from 37 to 44, and severe level of fatigue from 45 to 63.

Tool IV: The PROMIS Parent Proxy Short Form–Sleep Disturbance 8a is an 8-item parent-reported instrument used to assess sleep quality and sleep-related problems in children and adolescents over the previous seven days. In this study, sleep disturbance was assessed using the PROMIS Parent Proxy Short Form–Sleep Disturbance 8a, which was completed by the parent/caregiver to evaluate the child's sleep-related difficulties and nighttime behaviors from the caregiver's perspective. The use of a parent-proxy measure provides information on observable sleep characteristics that may not be fully captured through child self-report.

The instrument includes eight items assessing different aspects of sleep disturbance, including sleep quality, sleep initiation, and sleep continuity. Each item is rated on a 5-point Likert scale (1 = never, 2 = rarely, 3 = sometimes, 4 = often, and 5 = always), with total scores ranging from 8 to 40. Higher scores indicate greater sleep disturbance and poorer sleep quality (28). For interpretation, scores were categorized as follows: good sleep quality (<60% of the maximum score), fair sleep quality (60% to <80%), and poor sleep quality (80%–100%).

#### Validity and reliability of tools

Although the adopted instruments have been previously validated and widely used, their validity and reliability were reassessed in the current study to confirm their suitability within the specific population of children undergoing hematopoietic stem cell transplantation and the study context. Five pediatric nursing experts reviewed the tools to assess content validity, focusing on clarity, relevance, and appropriateness of the items.

Internal consistency reliability was reassessed in the current sample using Cronbach's alpha coefficients to ensure the consistency of the instruments when applied among children undergoing hematopoietic stem cell transplantation. The reliability coefficients were 0.86, 0.87, and 0.84 for tool II, tool III, and tool IV.

#### Pilot study

A pilot study was conducted with 10% of the calculated sample size (9 children) before the commencement of the main study to assess the feasibility of the study procedures, clarity and applicability of the data collection tools, estimated time required for data collection, and the practicality of implementing the intervention protocols. The pilot study participants were not excluded from the final sample because no modifications were required in the study tools, intervention procedures, or data collection process following the pilot phase. Therefore, their data were included in the final analysis.

### Ethical considerations

The study was conducted in accordance with the ethical principles outlined in the Declaration of Helsinki for research involving human participants. Ethical approval was obtained from the Scientific Research Ethics Committee of the Faculty of Nursing, Tanta University (Code No: 370-1-2024). Before participation, written informed consent was obtained from the parents or legal guardians of all enrolled children after providing a clear explanation of the study purpose, procedures, potential benefits, and possible risks. The researchers emphasized that participation was voluntary and that participants had the right to refuse participation or withdraw from the study at any time without affecting the care they received. Confidentiality and privacy of participants were strictly maintained throughout the study, and all collected data were handled anonymously and used solely for research purposes.

### Data collection

The researchers obtained the necessary official permissions for data collection by submitting an official letter issued by the Dean of the Faculty of Nursing to the directors of the study setting. Data were collected over nine months, from the beginning of August 2024 to the end of April 2025. The researchers attended the study setting every day of the week, from Saturday to Friday, between 9:00 am and 12:00 pm. During the intervention period, participants' attendance and completion of the scheduled intervention sessions were monitored using attendance records to ensure adherence to the planned intervention protocol. The researchers also ensured that the interventions were delivered according to the standardized protocol throughout the study period.

### The study was conducted through the following phases

#### Preparation

A thorough review of recent literature was conducted to develop the data collection tools and intervention protocols. The mindfulness intervention consisted of mindful breathing exercises. All mindfulness sessions were guided by trained researchers (two authors and six co-authors) who had received mindfulness training from a specialist in the Department of Psychiatry at Tanta University Hospitals. The intervention team was supervised by a mindfulness-trained researcher, an experienced physician with expertise in mindfulness techniques.

The researcher responsible for acupoint stimulation received specialized training in acupressure application for three months under the supervision of a specialist in physical medicine, rheumatology, and rehabilitation to ensure accurate identification and appropriate stimulation of the selected acupoints. The three acupoints selected for this study were Yintang (EX-HN3), located between the eyebrows and the nasal root; Zusanli (ST36), located on the anterolateral side of the leg, one fingerbreadth (middle finger) lateral to the anterior crest of the tibia; and Shenmen (HT7), located at the wrist crease on the radial side of the flexor carpi ulnaris tendon between the ulna and pisiform bones (29, 30) (Figure 2).

**Figure 2:**
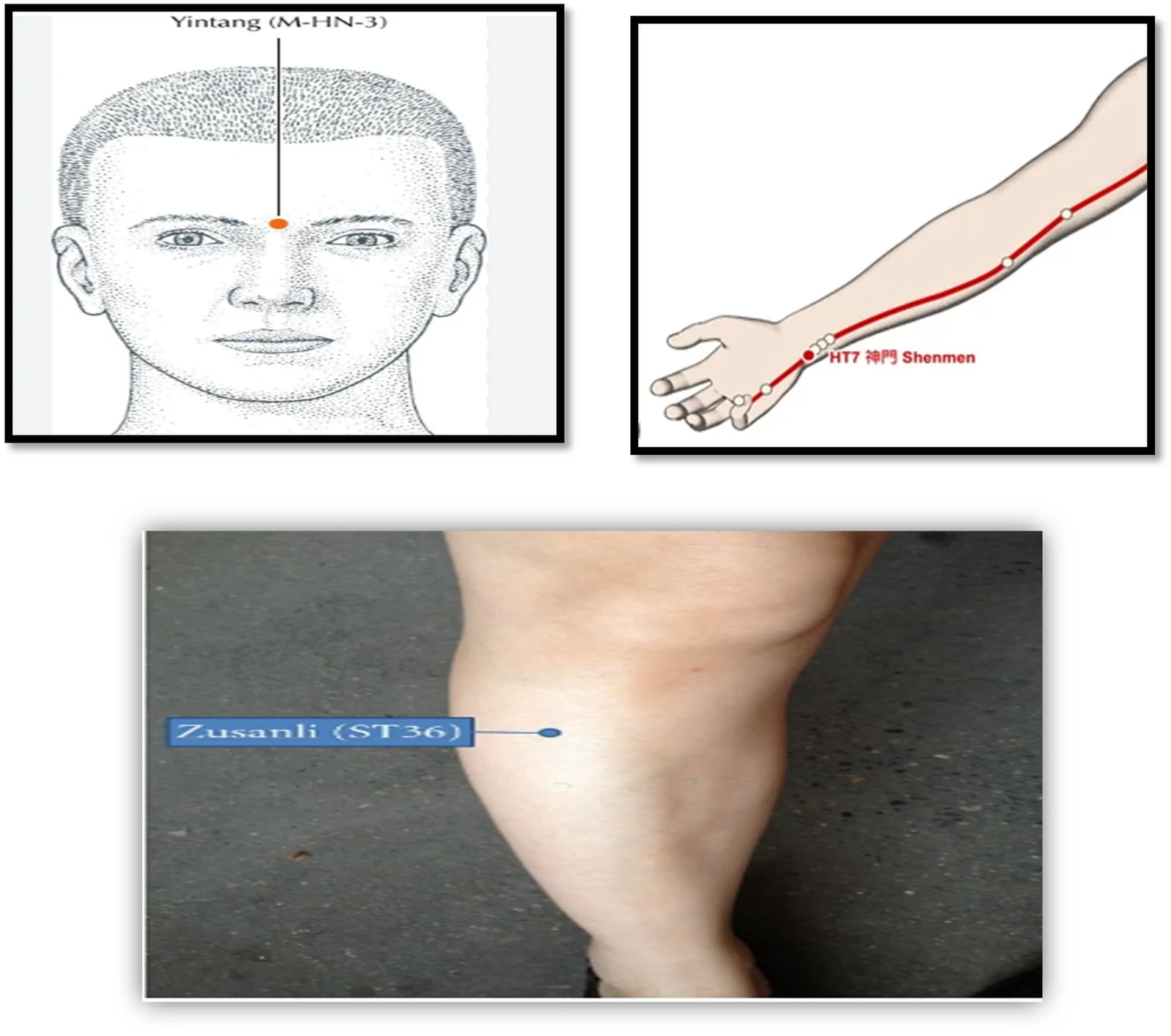
Locations of yintang (EX-HN 3), shenmen (HT 7), and zusanli (ST 36) acupoints.

#### Assessment

Initially, the researchers introduced themselves to the children and explained the purpose and procedures of the study. Each child was interviewed individually using Tool I to collect biopsychosocial and clinical data.

Before implementing mindfulness breathing and/or acupoint stimulation, baseline measurements of physiological parameters, anxiety level, fatigue severity, and sleep quality were obtained for all participants (pretest assessment).

### Implementation

#### Control group

Children in the control group received routine care according to the standard protocol of the stem cell transplantation unit. Routine care included the usual medical and nursing management provided to all children during the transplantation process, without receiving any additional structured intervention targeting anxiety reduction, fatigue management, or sleep improvement.

#### Mindfulness breathing group

Children in the mindfulness breathing group received routine care in addition to a 20-minute mindful breathing session once daily in the morning for seven consecutive days before stem cell transplantation during the chemotherapy preparation period. Sessions were conducted in the presence of the researchers and caregivers. The same intervention schedule was continued for one month after transplantation.

Each mindful breathing session consisted of four sequential steps, with each step lasting approximately five minutes:

Step 1: Identifying inhalation and exhalation

The child was guided to sit comfortably, gently close his/her eyes, take two slow deep breaths, and then return to natural breathing. The child was instructed to notice the airflow through the nose and focus attention on the breathing process.

Step 2: Following the entire length of the breath

The child was instructed to maintain a relaxed posture and focus on the complete cycle of breathing, including the beginning, middle, and end of inhalation and exhalation. If distractions occurred, such as sounds, bodily sensations, thoughts, or emotions, attention was gently redirected back to the breath.

Step 3: Connecting the mind and body

The child was guided to become aware of the connection between breathing and bodily sensations, recognizing the movement of the body during inhalation and exhalation and developing awareness of the body as a whole.

Step 4: Body relaxation

The child was guided to identify areas of tension and gradually release tension from different body regions, including the head, face, neck, arms, hands, chest, abdomen, legs, and feet. Finally, the child practiced whole-body relaxation through slow breathing until the session ended. At the end of each session, the researcher guided the child to gradually expand awareness to the surrounding environment, open his/her eyes, and return to full alertness.

#### Acupoint stimulation group

Children in the acupoint stimulation group received routine care in addition to finger acupressure sessions once daily for seven consecutive days before stem cell transplantation. The sessions continued using the same schedule for one month after transplantation (31, 32).

The acupressure procedure was performed as follows:

- The intervention environment was prepared by ensuring a warm, quiet, and well-ventilated room, and children were positioned comfortably.
- The researcher maintained appropriate hygiene by keeping nails short and clean, removing rings, washing and warming hands before the procedure, and maintaining a comfortable position during application.
- Pressure was applied for 15 min to the three selected acupoints (Yintang, Zusanli, and Shenmen), with five minutes allocated for each point. The researcher used the thumb in a rotating motion at approximately 20–25 cycles per minute with light-to-moderate pressure, sufficient to produce a mild sensation without causing discomfort.

Due to the nature of the interventions, blinding of participants and intervention providers was not feasible. However, outcome assessors who were not involved in intervention delivery performed the outcome assessments.

### Intervention adherence and fidelity

All participants completed the planned intervention sessions during the one-month intervention period. Attendance was recorded throughout the intervention period to monitor adherence to the intervention protocol. Mindfulness breathing and acupoint stimulation were delivered according to standardized protocols to ensure consistency of intervention implementation across participants.

### Evaluation phase

Physiological parameters, anxiety level, fatigue severity, and sleep quality were reassessed at two time points: one week after the intervention (the seventh day, before stem cell transplantation during the preparation regimen) and one month after transplantation during the early post-transplantation phase. Outcomes were assessed for all three groups, and comparisons among groups were performed using appropriate statistical analyses.

### Statistical analysis

Data were analyzed using the Statistical Package for the Social Sciences (SPSS) version 26. Continuous variables were presented as mean ± standard deviation (SD), while categorical variables were presented as frequencies and percentages. We assessed the normality of continuous variables using the Shapiro–Wilk test, supported by evaluation of skewness and kurtosis values and visual inspection of histograms and Q–Q plots. Variables showing an approximately normal distribution were analyzed using parametric tests.

Although the sample size estimation was based on between-group comparisons, the longitudinal analysis incorporated repeated measurements from the same participants, allowing assessment of temporal changes and interaction effects while accounting for within-subject correlation. The Group×Time interaction was considered the primary indicator of differential intervention effects over time among the three groups.

We compared baseline socio-demographic and biomedical characteristics among the three studied groups to assess group homogeneity. Categorical variables were compared using the Pearson chi-square test (or Fisher's exact test when appropriate). We compared continuous variables, such as age and duration of hospitalization, using one-way analysis of variance (ANOVA). For physiological parameters (Table 1) and psychological outcomes, including anxiety, fatigue severity, and sleep disturbance scores (Table 2), we analyzed repeated measurements using repeated-measures ANOVA/linear mixed models to assess the effects of group, time, and the group×time interaction. The Group×Time interaction was considered the main indicator of whether the intervention effects differed among the study groups over time.

**Table 1:** Comparison of physiological parameters among groups over time with *post hoc* analysis (*n* = 90).

Physiological parameters	Time points	Control Mea*n* ± SD	Mindfulness Mean ± SD	Acupoint stimulation Mean ± SD	Between-group *p*-value	Group×Time interaction	Effect size (Partial *η*^2^)	95% CI	Post hoc comparison
Systolic blood pressure (mmHg)	Baseline	121.50 ± 6.96	122.33 ± 7.62	121.63 ± 7.09	0.982	0.927	0.01	0.00–0.04	No significant differences
	One week after intervention	121.00 ± 6.48	122.33 ± 7.62	120.97 ± 6.46	0.682				
	One month after intervention	120.63 ± 6.21	120.20 ± 6.33	120.10 ± 6.01	0.939				
Diastolic blood pressure (mmHg)	Baseline	83.73 ± 5.30	82.67 ± 6.39	82.00 ± 7.14	0.566	<0.001*	0.24	0.13–0.34	No significant differences
	One week after intervention	82.53 ± 4.97	76.67 ± 8.02	76.00 ± 8.13	0.001*				Mindfulness vs. Control*; Acupoint vs. Control*
	One month after intervention	81.40 ± 3.53	71.00 ± 7.92	76.67 ± 4.79	<0.001*				Mindfulness vs. Control*; Acupoint vs. Control*; Mindfulness vs. Acupoint*
Respiratory rate (breaths/min)	Baseline	27.40 ± 5.39	28.50 ± 4.78	26.37 ± 2.29	0.173	<0.001*	0.39	0.28–0.48	No significant differences
	One week after intervention	29.10 ± 5.25	23.00 ± 1.23	23.23 ± 1.40	<0.001*				Mindfulness vs. Control*; Acupoint vs. Control*
	One month after intervention	28.90 ± 4.05	17.07 ± 2.44	18.67 ± 2.24	<0.001*				Mindfulness vs. Control*; Acupoint vs. Control*; Mindfulness vs. Acupoint*
Pulse rate (beats/min)	Baseline	104.07 ± 4.44	106.67 ± 4.45	105.93 ± 4.54	0.074	<0.001*	0.32	0.20–0.42	No significant differences
	One week after intervention	103.33 ± 6.23	95.53 ± 5.99	98.53 ± 4.31	<0.001*				Mindfulness vs. Control*; Acupoint vs. Control*
	One month after intervention	101.87 ± 2.90	92.00 ± 7.76	95.67 ± 3.85	<0.001*				Mindfulness vs. Control*; Acupoint vs. Control*; Mindfulness vs. Acupoint*

*indicates a *p*-value < 0.05 (statistically significant).

**Table 2:** Comparison of anxiety, fatigue severity, and sleep disturbance scores among the studied groups over time with *post hoc*.

Variables	Time points	Control group (*n* = 30) Mean ± SD	Mindfulness group (*n* = 30) Mean ± SD	Acupoint stimulation group (*n* = 30) Mean ± SD	Between-group comparison (*p*-value)	Group×Time interaction (*p*-value)	Effect size (Partial η^2^)	95% CI	Post hoc comparison
State-Trait Anxiety Inventory for Children	Baseline	42.40 ± 3.23	42.93 ± 5.83	44.80 ± 5.03	0.057	<0.001*	0.31	0.19–0.41	No significant differences
	One week after intervention	39.67 ± 4.66	35.60 ± 4.33	36.93 ± 4.51	0.003*				Mindfulness < Acupoint stimulation < Control
	One month after intervention	37.07 ± 6.51	27.80 ± 3.88	30.20 ± 4.35	<0.001*				Mindfulness < Acupoint stimulation < Control
Fatigue Severity Scale	Baseline	47.67 ± 6.64	48.00 ± 5.58	47.93 ± 5.61	0.990	<0.001*	0.28	0.16–0.39	No significant differences
	One week after intervention	41.87 ± 4.52	38.13 ± 4.55	39.27 ± 5.29	0.005*				Mindfulness < Acupoint stimulation < Control
	One month after intervention	38.47 ± 5.70	32.27 ± 5.02	34.07 ± 3.83	<0.001*				Mindfulness < Acupoint stimulation < Control
PROMIS Parent Proxy Short Form–Sleep Disturbance 8a	Baseline	37.40 ± 2.19	36.40 ± 2.59	36.27 ± 2.91	0.349	<0.001*	0.35	0.23–0.45	No significant differences
	One week after intervention	35.53 ± 3.36	27.63 ± 3.77	29.07 ± 3.72	<0.001*				Mindfulness < Acupoint stimulation < Control
	One month after intervention	32.73 ± 5.19	21.20 ± 2.41	26.00 ± 2.29	<0.001*				Mindfulness < Acupoint stimulation < Control

Between-group comparisons at each time point were performed using one-way analysis of variance (ANOVA), followed by Bonferroni *post hoc* tests for multiple comparisons. We analyzed longitudinal changes among groups using repeated-measures ANOVA/linear mixed models to assess the group×time interaction. *p < 0.05.

At each assessment point (baseline, one week after intervention, and one month after intervention), differences among the three groups were examined using one-way ANOVA. Significant ANOVA results were followed by Bonferroni-adjusted *post hoc* pairwise comparisons to determine differences between specific groups (mindfulness vs. control, acupoint stimulation vs. control, and mindfulness vs. acupoint stimulation). All statistical tests were two-tailed, and statistical significance was set at *p* < 0.05. Values of *p* < 0.001 were considered highly statistically significant (33).

## Results

The sociodemographic characteristics of the studied children are presented in Table 3. The mean age of children was comparable among the three groups (11.13 ± 2.58 years in the control group, 11.76 ± 3.53 years in the mindfulness breathing group, and 12.20 ± 3.22 years in the acupoint stimulation group). The distribution of children according to age category, gender, residence, educational status, and birth order showed no statistically significant differences among the three groups (*p* > 0.05), indicating baseline comparability of sociodemographic characteristics.

**Table 3:** Percentage distribution of socio-demographic data of the studied children (*n* = 90).

Children's socio-demographic data	Control group (*n* = 30)		Mindfulness group (*n* = 30)		Acupoint stimulation group (*n* = 30)		*χ* 2 P
	No	%	No	%	No	%	
Age (years):							
7–11	16	53.3	19	63.3	14	46.7	1.702 0.427
12–17	14	53.3	11	36.7	16	53.3	
Mean ± SD	11.13 ± 2.58		11.76 ± 3.53		12.20 ± 3.22		
Gender							
Male	18	60.0	14	46.7	16	53.3	1.071 0.585
Female	12	40.0	16	53.3	14	46.7	
Residence:							
Rural	14	46.7	14	46.7	22	73.3	5.760 0.056
Urban	16	53.3	16	53.3	8	26.7	
Educational status:							
Illiterate	2	6.7	3	10.0	3	10.0	0.044 0.978
Elementary school	23	76.7	22	73.3	21	70.0	
Secondary school	5	16.6	5	16.7	6	20.0	
Birth order							
First	20	66.7	18	60.0	12	40.0	10.880 0.092
Second	10	33.3	8	13.3	12	40.0	
Third	0	0.0	0	0.0	4	13.3	
Fourth	0	0.0	0	0.0	2	6.7	

Pearson chi-square; S.D., standard deviation.

Table 4 presents the biomedical characteristics of the studied children. The three groups were comparable regarding medical diagnosis, with leukemia being the most common diagnosis across groups. Most children were undergoing their first transplantation, and the mean duration of hospitalization was similar among the control, mindfulness breathing, and acupoint stimulation groups (43.27 ± 2.18, 43.13 ± 2.40, and 43.13 ± 2.40 days, respectively). No statistically significant differences were observed among groups regarding transplant number, transplant type, stem cell source, or conditioning regimen (*p* > 0.05), confirming baseline clinical homogeneity before intervention.

**Table 4:** Percentage distribution of biomedical data of the studied children (*n* = 90).

Children's socio-demographic data	Control group (*n* = 30)		Mindfulness group (*n* = 30)		Acupoint stimulation group (*n* = 30)		χ 2 P
	No	%	No	%	No	%	
Medical Diagnosis							
Leukemia (ALL)	10	33.3	11	36.7	10	33.3	1.727
Burkitt lymphoma	8	26.7	7	23.3	8	26.7	0.422
Hodgkin lymphoma	4	13.3	6	20.0	4	13.3	
Beta thalassemia	4	13.3	4	13.3	5	16.7	
Neuroblastoma	4	13.3	2	6.7	3	10.0	
Duration of Hospitalization (in days) Mean ± SD	43.27 ± 2.18		43.13 ± 2.40		43.13 ± 2.40		F: 0.325 0.850
Transplant number							2.00 0.368
1st transplant	28	93.3	29	96.7	29	96.7	
2nd transplant	2	6.7	1	3.3	1	3.3	
3rd transplant	0	0.0	0	0.0	0	0.0	
Transplant type							
Autologous	16	53.3	12	40.0	14	46.7	1.333
Allogeneic	14	46.7	18	60.0	16	53.3	0.513
Stem cell source							0.250
Bone marrow	6	20.0	5	16.7	6	20.0	
Peripheral blood	24	80.0	25	83.3	24	80.0	0.882
Preparation regimen							
Myeloablative	30	100.0	30	100.0	30	100.0	-
Reduce intensity							

Pearson chi-square; S.D., standard deviation.

Regarding physiological parameters (Table 1), significant between-group differences were observed in diastolic blood pressure, respiratory rate, and pulse rate following the interventions. One month after the intervention, the mindfulness group demonstrated a reduction of 10.40 mmHg in diastolic blood pressure compared with the control group, while the acupoint stimulation group showed a reduction of 4.73 mmHg. Additionally, the mindfulness group showed a significantly greater reduction in diastolic blood pressure compared with the acupoint stimulation group (mean difference = 5.67 mmHg). For respiratory rate, the mindfulness and acupoint stimulation groups exhibited clinically meaningful reductions compared with the control group at one month after intervention, with mean differences of 11.83 and 10.23 breaths/min, respectively. The mindfulness group also demonstrated a greater reduction compared with the acupoint stimulation group (mean difference = 1.60 breaths/min).

Similarly, pulse rate decreased significantly in both intervention groups compared with the control group at one month after intervention. The mindfulness group showed a reduction of 9.87 beats/min compared with the control group, whereas the acupoint stimulation group showed a reduction of 6.20 beats/min. The mindfulness group also demonstrated a greater reduction compared with the acupoint stimulation group (mean difference = 3.67 beats/min). These findings indicate that the observed statistically significant effects were accompanied by meaningful changes in physiological parameters.

As presented in Table 2, both intervention groups demonstrated significant improvements in anxiety, fatigue severity, and sleep disturbance scores compared with the control group over time. One month after intervention, children in the mindfulness group showed a reduction of 9.27 points in anxiety scores compared with the control group, while the acupoint stimulation group showed a reduction of 6.87 points. Furthermore, the mindfulness group demonstrated a greater reduction in anxiety scores compared with the acupoint stimulation group (mean difference = 2.40 points).

Regarding fatigue severity, the mindfulness group showed a reduction of 6.20 points compared with the control group, while the acupoint stimulation group showed a reduction of 4.40 points. The difference between the mindfulness and acupoint stimulation groups was 1.80 points, favoring the mindfulness intervention. For sleep disturbance, the mindfulness group demonstrated a reduction of 11.53 points compared with the control group, whereas the acupoint stimulation group showed a reduction of 6.73 points. The mindfulness intervention also resulted in a greater improvement compared with acupoint stimulation, with a mean difference of 4.80 points. These findings suggest that the statistically significant differences observed were associated with substantial improvements in psychological symptoms and sleep-related outcomes.

Table 5 Mindfulness group (Group I): Showed a gradual reduction in anxiety and fatigue, with notable improvement in sleep quality. By one month, 80% reported adequate sleep, and mild fatigue increased (80%). Anxiety decreased from predominantly above-average/high levels to mostly low/average (66.7% low/average). The acupoint stimulation group (Group II) demonstrated the fastest and most significant improvement. After one month, the majority had low anxiety (66.7%) and mild fatigue (80%). Sleep quality improved markedly, with 80% reporting fair to good sleep.

**Table 5:** Distribution of the studied children in each group according to their anxiety, fatigue severity levels, and sleep quality throughout the periods of intervention (*n* = 90).

Variables	Control group (*n* = 30)						Mindfulness group (*n* = 30)						Acupoint stimulation (*n* = 30)					
	Before intervention		One week of intervention		One month after intervention		Before intervention		One week of intervention		One month after intervention		Before intervention		One week of intervention		One month after intervention	
	N %		N %		N %		N %		N %		N %		N %		N %		N %	
Anxiety levels																		
Low	0	0	2	6.7	6	20	2	6.7	4	13.3	26	86.7	0	0.0	4	13.3	20	66.7
Average	15	50	18	60	14	46.7	6	20	24	80	4	13.3	4	13.3	20	66.7	10	33.3
Above average	15	50	10	33.3	10	33.3	20	66.6	2	6.7	0	0.0	22	73.4	6	20	0	0.0
Very high	0	0.0	0	0.0	0	0.0	2	6.7	0	0.0	0	0.0	4	13.3	0	0.0	0	0
Fatigue levels																		
Mild	0	0.0	2	6.7	6	20.0	0	0.0	9	30.0	24	80.0	0	0.0	7	23.3	24	80.0
Moderate	10	33.3	16	53.3	18	60.0	8	26.7	18	60.0	4	13.3	8	26.7	19	63.4	6	20.0
Severe	20	66.7	12	40	6	20.0	22	73.3	3	10.0	2	6.7	22	73.3	4	13.3	0	0.0
Sleep quality																		
Good	0	0	0.0	0.0	3	10.0	0	0.0	2	6.7	26	86.7	0	0.0	2	6.7	6	20.0
Fair	0	0	4	13.3	6	20.0	0	0.0	22	73.3	4	13.3	2	6.7	18	60.0	24	80.0
Poor	30	100	26	86.7	21	70.0	30	100.0	6	20.0	0	0.0	28	93.3	10	33.3	0	0.0

Taken together, these findings supported both research hypotheses. The first hypothesis (H1) was supported, as children who received either mindfulness breathing or acupoint stimulation demonstrated significantly lower anxiety, fatigue severity, and sleep disturbance scores compared with those receiving routine care. The second hypothesis (H2) was also supported, as mindfulness breathing produced greater improvements in anxiety, fatigue severity, and sleep disturbance outcomes than acupoint stimulation and routine care, particularly at the one-month assessment.

## Discussion

Children undergoing hematopoietic stem cell transplantation (HSCT) are vulnerable to a wide range of physiological and psychological challenges, including pain, fatigue, sleep disturbances, anxiety, and stress, which may negatively affect their recovery and quality of life. Therefore, identifying safe and effective supportive interventions is essential to alleviate these treatment-related burdens (34).

The present randomized controlled trial demonstrated that both mindfulness breathing and acupoint stimulation were effective complementary interventions for improving physiological and psychological outcomes among children undergoing hematopoietic stem cell transplantation (HSCT). Compared with routine care, both intervention groups showed significant improvements in physiological parameters, anxiety, fatigue severity, and sleep disturbance after one month. The magnitude of these effects was supported by the observed effect sizes and confidence intervals, indicating meaningful differences beyond statistical significance. Although both interventions were beneficial, mindfulness breathing generally demonstrated greater improvements across the assessed outcomes compared with acupoint stimulation.

Regarding physiological parameters, the current study revealed that children who received mindfulness breathing had the greatest reductions in diastolic blood pressure, respiratory rate, and pulse rate after one month of intervention compared with both the acupoint stimulation and control groups. These findings suggest that mindfulness breathing may effectively reduce physiological stress responses through enhancement of relaxation and emotional regulation. Anxiety and psychological stress can activate the sympathetic nervous system, resulting in increased secretion of stress-related hormones, including epinephrine, norepinephrine, and cortisol, which subsequently contribute to elevations in blood pressure, heart rate, and respiratory rate (35). Therefore, reducing anxiety and promoting relaxation through mindfulness practices may contribute to improved physiological stability. These findings are consistent with Dietrich and Bidart (2024) (36), who reported that a 20-minute mindfulness intervention significantly reduced respiration rate and heart rate among female students.

The acupoint stimulation group also demonstrated significant improvements in diastolic blood pressure, respiratory rate, and pulse rate compared with routine care; however, the magnitude of improvement was lower than that observed in the mindfulness breathing group. These findings are supported by Nadali et al. (2024) (23), who reported significant reductions in blood pressure, respiratory rate, and heart rate following acupressure applied to the Yintang point. Similarly, Bal and Gun (2024) (37) found significant decreases in blood pressure, heart rate, and respiratory rate among patients undergoing coronary angiography following acupressure intervention. However, these results differ from those of Koç and Balcı (2020) (31), who reported no significant changes in heart rate and oxygen saturation following acupressure applied to LI4, L11, and HT7 points among pediatric patients. These variations may be attributed to differences in sample characteristics, clinical conditions, cultural context, stimulated acupoints, duration and intensity of pressure application, and timing of outcome assessment. Overall, the present findings indicate that both mindfulness breathing and acupoint stimulation were more effective than routine care in improving physiological stress indicators, with mindfulness breathing showing a greater effect.

The current study also demonstrated that children receiving either mindfulness breathing or acupoint stimulation experienced significantly lower levels of anxiety, fatigue severity, and sleep disturbance compared with the control group after one month of intervention. The beneficial effects of mindfulness breathing may be explained by its role in enhancing emotional regulation and reducing stress responses through modulation of brain regions involved in cognitive control and emotional processing, including the prefrontal cortex and amygdala (5). By improving awareness of thoughts and emotions, mindfulness may help children develop more effective coping strategies for the psychological challenges associated with HSCT. These findings are consistent with Komariah et al. (2022) (38), who reported that four weeks of mindfulness breathing practice reduced stress and anxiety among university students. Similarly, Ng et al. (2021) (15) demonstrated that a single 30-minute mindful breathing session was effective in reducing fatigue among patients with hematological cancer. In addition, a study conducted in Malaysia reported that 20 min of mindful breathing was beneficial in reducing anxiety among patients receiving palliative care (39).

Furthermore, previous studies have supported the effectiveness of mindfulness-based interventions in improving psychological outcomes among patients with cancer. A 20-minute mindfulness breathing intervention was shown to reduce anxiety among cancer patients (40). Mindfulness interventions have also been increasingly recognized as effective approaches for improving anxiety, managing fatigue, and enhancing positive affect among patients during the early post-transplant period (10). Moreover, mindfulness practice has been associated with improved emotional self-regulation and reduced stress and anxiety symptoms (41).

The acupoint stimulation group similarly showed improvements in anxiety, fatigue, and sleep disturbance outcomes. These findings are consistent with Salajegheh et al. (2024) (20), who reported that acupressure improved sleep quality and reduced anxiety among burn patients. Additionally, Bal and Gun (2024) (37) demonstrated that acupressure reduced state anxiety and pain among patients undergoing coronary angiography. The beneficial effects of acupressure may be related to its ability to regulate autonomic nervous system activity by reducing sympathetic activation and enhancing parasympathetic responses (32). Furthermore, acupressure may influence neurotransmitters and hormones involved in stress regulation and sleep processes, including melatonin, serotonin, adrenocorticotropic hormone, and endorphins, thereby contributing to improved sleep quality and reduced anxiety symptoms (42).

Sleep disturbance represents an important concern among children undergoing HSCT because it may negatively affect emotional well-being, recovery, and overall quality of life. The present study demonstrated significant improvement in sleep disturbance scores among children receiving mindfulness breathing and acupoint stimulation, with the mindfulness group showing the greatest improvement after one month. This finding suggests that mindfulness breathing may be particularly effective in promoting relaxation and improving sleep-related outcomes during the transplantation period. These findings are supported by Garcia et al. (2025) (43), who reported that mindfulness interventions reduced stress and anxiety while improving sleep quality. Similarly, Compernolle and Sled (2020) (13) found that mindfulness practice improved relaxation and sleep quality after two weeks of intervention.

Overall, the study findings provide support for both proposed research hypotheses. The significant improvements in anxiety, fatigue severity, and sleep disturbance among children receiving mindfulness breathing or acupoint stimulation compared with routine care support H1. Furthermore, the greater improvements observed in the mindfulness breathing group across these outcomes support H2, suggesting that mindfulness breathing may provide greater benefits than acupoint stimulation for managing these symptoms during the HSCT period. These findings should, however, be interpreted within the context of the study's sample size, single-center design, and relatively short follow-up period.

## Conclusion

This randomized controlled trial demonstrated that mindfulness breathing and acupoint stimulation are promising non-pharmacological interventions for improving physiological and psychological outcomes among children undergoing hematopoietic stem cell transplantation. Both interventions were associated with significant improvements in anxiety, fatigue severity, sleep disturbance, and selected physiological parameters compared with routine care, with mindfulness breathing showing greater improvements across several outcomes. These findings highlight the potential role of complementary nursing interventions in supporting symptom management and enhancing the well-being of pediatric HSCT recipients. Future multicenter studies with larger samples, longer follow-up periods, and objective fidelity assessment are recommended to further evaluate the sustained effects and applicability of these interventions in diverse pediatric HSCT populations.

### Limitations

This study has several limitations that should be considered when interpreting the findings. First, it was conducted in a single facility with a relatively small sample size, which may limit the generalizability of the results to other pediatric HSCT populations. Second, blinding of participants and intervention providers was not feasible due to the nature of the interventions, which may have introduced potential response bias, particularly for self-reported outcomes and parent-reported measures. Third, the follow-up period was limited to one month after the intervention, preventing assessment of the long-term sustainability of the intervention effects. Additionally, objective fidelity monitoring of intervention delivery was not performed, which may have limited the evaluation of intervention consistency. Fourth, other factors related to the HSCT process, such as treatment intensity, hospitalization-related stress, family support, and additional supportive care, could not be completely controlled and may have influenced the observed outcomes. Future studies should consider larger multicenter designs, longer follow-up periods, objective fidelity assessments, and investigation of the combined effects of mindfulness breathing and acupoint stimulation.

Also, a potential limitation of this study is the use of different informants across outcome measures. While anxiety and fatigue outcomes were assessed using child-reported measures, sleep disturbance was evaluated using a parent-proxy instrument (PROMIS Parent Proxy Short Form–Sleep Disturbance 8a). Parent and child reports may reflect different perspectives, as children may provide insight into subjective experiences such as emotional symptoms and fatigue, whereas parents may provide more reliable observations of sleep patterns and behaviors. Therefore, differences in informant sources should be considered when interpreting comparisons across outcomes. Future studies may benefit from incorporating both child-reported and parent-reported measures to provide a more comprehensive assessment of these outcomes.

Although all participants completed the scheduled intervention sessions, objective fidelity assessment was not performed. The use of structured fidelity assessment tools, intervention checklists, or independent monitoring in future studies may provide a more comprehensive evaluation of intervention delivery consistency.

## Data Availability

The original contributions presented in the study are included in the article/Supplementary Material, further inquiries can be directed to the corresponding author.
